# Exploring internet addiction during Covid‐19 pandemic: a comparative study considering psychological, social, familial and individual aspects in University students

**DOI:** 10.1002/hsr2.70118

**Published:** 2024-10-15

**Authors:** Mahbobeh Faramarzi, Bardia Karim, Zeinab Gholami, Fatemah Khoshkhou, Fatemeh Shirazi, Negar Soghli, Munire Parvaneh, Hoda Shirafkan, Faezeh Khorshidian

**Affiliations:** ^1^ Social Determinants of Health Research Center, Health Research Institute Babol University of Medical Sciences Babol Iran; ^2^ Health Research Institute Babol University of Medical Sciences Babol Iran; ^3^ Department of Community Medicine, School of Medicine, Social Determinants, Health Research Institute Babol University of Medical Sciences Babol Iran; ^4^ Department of Psychiatry, School of Medicine, Health Research Institute Babol University of Medical Sciences Babol Iran

**Keywords:** addictive behaviors, COVID‐19, internet addiction, university students

## Abstract

**Background:**

Internet addiction causes a variety of social, interpersonal, psychological, and physical issues. We are confronting a worldwide crisis about internet addiction and its effects.

**Objectives:**

Utilizing five questionnaires to survey university students during the COVID‐19 epidemic, the current study sought to evaluate internet addiction and associated risk variables.

**Methods:**

At the University of Medical Sciences in North Iran, a cross‐sectional analytical investigation was carried out. The sample was done among 318 students, selected through the random cluster sampling method. Data collection was done during August and September 2021.

**Results:**

Based on our findings, the prevalence of internet addiction was 28.9% (92/318). Our results revealed that women are at twice the twice the risk of internet addiction as men (*p* = 0.028). Students in the medicinal field (*p* = 0.043; students with a positive history of mental illness in the family) *p* = 0.001 (and students with a fear of COVID‐19) *p* = 0.002 (recognized in the risk of Internet addiction, thus depression) *p* < 0.001 (anxiety) *p* < 0.001 (somatization) *p* = 0.001 (and psychological distress) *p* < 0.001 are risk factors for internet addiction. Age, marital status, place of living, total social support, and use of Alcohol/cigarettes are not risk factors for internet addiction.

**Conclusions:**

The findings suggest that university students with a fear of COVID‐19, depression, anxiety, somatization, psychological distress and a positive history of mental illness in the family, particularly medicinal field students, are at significant risk for Internet addiction.

## 1 BACKGROUND

The use of the Internet as a part of people's daily lives is increasing in different societies around the world, even in less developed countries.^1^, ^2^, ^3^, ^4^ In today's world, the Internet facilitates interpersonal communication and business transactions and provides the possibility of providing services in various fields. Even today, using the Internet, the possibility of telemedicine and telepsychiatry has been provided.^1^, ^5^

Thanks to extensive study, Internet use disorder was classified as an addictive illness by the American Psychiatric Association when it was first added to the fifth edition of the Diagnostic and Statistical Manual of Mental Disorders’ Appendix.^6^

The DSM 5 appendix of impulse control disorders provides a definition and description of internet addiction. The DSM‐5 diagnostic criteria^7^ state that the following are warning indicators of Internet addiction (IA): persistent fixation with the Internet and recurrent thoughts about one's past or upcoming online behavior; To feel satisfied, the person is steadily increasing the amount of time they spend on the internet. Repeatedly and unsuccessfully trying to reduce the time spent using the Internet or stop it; Experiences agitation, dysphoria, and even irritability and depression; Staying online for longer than originally planned Following the constant use of the Internet, which affects various aspects of a person's life, including important social relationships, a person's occupational and academic performance, and even endangering the person; Repeated lying to others and even to the therapist to keep the internet usage secret; urging the Internet to suppress unpleasant emotional feelings such as sadness, depression, anxiety, hopelessness, and guilt; Being guarded and defensive about the issue of constant use of the Internet; A feeling of freshness and euphoria when using and being active on the Internet; The development of physical symptoms due to Internet addiction.^6^, ^7^

There are five distinct categories of internet addiction: The phrase “internet addiction” is wide and encompasses a variety of behaviors and issues with impulse control related to using mobile, personal computers, and internet technology. Although there are no recognized standards for diagnosing online addiction, researchers have distinguished five subtypes of particular computer and internet addictions. Cyber‐Addiction: One of the easier online addictions to understand is cybersex. It includes adult websites, adult chat rooms with sexual fantasies, adult websites with pornography, and XXX webcam services.^7^, ^8^, ^9^

One's capacity to establish romantic, personal, or sexual connections in real life may suffer from a preoccupation with any one of these services. Addicts to cybersex can receive treatment; this usually consists of intervention followed by continued inpatient or outpatient therapy. Net Compulsions: Compulsive online shopping, online gambling, stock trading, online auctions (like eBay), and trading are examples of interactive online activities that may be quite destructive. These behaviors may harm one's capacity to maintain financial security and interfere with work‐related responsibilities.^8^, ^9^, ^10^ Stress in relationships can also result from overspending or losing a lot of money. Those who are already prone to gambling or spending addictions might easily become hooked on the internet due to the quick and simple access to online casinos and shops. Cyber‐Addiction in Relationships: Addicts to cyberspace or online connections are engrossed in establishing and sustaining relationships virtually, frequently overlooking and undervaluing their in‐person friends and family. Online relationships may develop wherever individuals can communicate with one another online; however, they usually do so in chat rooms or on other social networking sites. People who pursue connections online sometimes do so while hiding their true identities and physical characteristics; this contemporary practice gave rise to the nickname “catfish.”.^9^, ^10^

Engaging excessively in an online social life and creating a virtual identity might result in a person having restricted social abilities and having unrealistic expectations when it comes to face‐to‐face encounters.^10^, ^11^ Oftentimes, this results in a lack of capacity to establish connections in the real world, thus increasing the individual's reliance on their online contacts. Typically, counseling or treatment is necessary to address this addiction and guarantee enduring modifications in behavior.^10^, ^11^, ^12^

Excessive and uncontrollable need to gather information: The internet offers people a plethora of information and expertise. For many individuals, the ease of accessing information has transformed into an insatiable need to collect and systematize data. Information‐seeking may often be a sign of pre‐existing obsessive‐compulsive tendencies.^9^, ^10^ An excessive and uncontrollable need for knowledge may also decrease work efficiency and ultimately result in job loss. The treatment options for addiction vary depending on its severity and might include various therapeutic techniques that aim to modify obsessive behavior and build coping mechanisms, as well as the use of medicines.^9^, ^10^, ^11^

Addiction to computers or gaming: Addiction to computers, often known as gaming addiction, encompasses both online and offline computer‐related behaviors.^9^, ^10^, ^11^, ^12^ As computers became more accessible, software was added to them to include games like Tetris, Minesweeper, and Solitaire. It did not take long for researchers to discover that compulsive computer game playing was becoming problematic in several situations. Playing these games for extended periods of time would lead to office workers becoming noticeably less productive. Thousands of new games have been released since the release of these original ones, and computer game addiction is still as common and dangerous as ever.^8^, ^9^, ^10^, ^11^

Gamers with gaming conditions are among the many forms of Internet addiction. According to ICD 11, gaming disorder is defined as a pattern of persistent or recurrent gaming behavior, also known as “digital gaming” or “video‐gaming,” that can occur offline or online. This behavior can take the following forms: 1. impaired control over gaming (pertaining to onset, frequency, intensity, duration, termination, and context); 2. Increasing priority given to gaming to the point where it trumps other interests and daily activities; and 3. continuation or escalation of gaming despite the occurrence of negative consequences.^13^, ^14^, ^15^

The gaming behavior pattern might be episodic, recurring, or continuous. Gamers who engage in this pattern of behavior have severe distress or major impairment in their ability to perform in essential areas such as family, work, education, and personal life. Before a diagnosis can be made, the gaming behavior and accompanying symptoms must typically be present for at least a year. However, if all diagnostic criteria are satisfied and the symptoms are severe, this time frame may be lowered. Among the necessary (essential) elements are:

- A consistent pattern of playing video games (also known as “digital gaming” or “video‐gaming”), which can take the form of offline or mostly online gaming (via the internet or other comparable electronic networks), exhibiting all of the following symptoms:

o Limited control over the course, frequency, intensity, length, termination, and context of gaming behavior;
o Giving gaming behavior a higher priority than other pursuits in life and everyday tasks to the point where gaming takes precedence.
o Persisting in or intensifying gaming habits in spite of unfavorable outcomes (such as family strife brought on by gaming, subpar academic achievement, or detrimental effects on health).

- The pattern of gaming behavior might be episodic, recurring, or continuous, but it always appears over a long period of time (like a year).
- The gaming behavior is neither caused by drug or substance side effects, nor is it better explained by another mental illness (such as a manic episode).
- The habit of gaming causes a great deal of distress or impairment in social, familial, professional, educational, or other crucial domains of functioning.^13^, ^14^, ^15^, ^16^, ^17^

According to the DSM‐5‐TR, an individual with IGD must experience “significant impairment or distress” across several domains of their life. Problems with general internet use, online gambling, social media use, or smartphone use are not included in this suggested diagnosis; instead, it is restricted to gaming. The following are some suggested signs of internet gaming disorder: obsession with gaming, withdrawal symptoms (sadness, anxiety, anger) when gaming is restricted or impossible, Tolerance, the desire to play games longer to satisfy the craving, being unable to cut back on gaming, fruitless attempts to stop playing, Giving up other pursuits, losing interest in past hobbies as a result of gaming, Playing the game in spite of issues, misleading relatives or other people about how much time is spent gaming, Playing video games to escape from depressing emotions like guilt or sorrow Danger of losing a career or a relationship as a result of gaming.^12^, ^13^, ^14^, ^15^, ^16^

Physical symptoms such as carpal tunnel syndrome, eyesight impairment and loss, back and neck pain, and disturbed sleep patterns can result from an addiction to the internet or computers. One of the major effects that we see today is its effect on people's nutrition and weight, which can create significant weight gain due to hypoactivity or using fast food, or, on the contrary, significant weight loss due to long hours of using the Internet and neglecting proper nutrition.^18^

We must acknowledge that IA causes issues in the areas of personal, familial, academic, financial, and professional life. Excessive use of the Internet leads to impairments of real‐life relationships, even between family members.^19^, ^20^, ^21^

One of the worst and most challenging consequences of Internet addiction is its impact on mental health, especially the occurrence of depression and anxiety symptoms. The issue of mental health and psychiatric disorders caused by the Internet in different communities has worried experts in this field, and many believe in proper screening and a timely treatment approach regarding this issue. It is necessary to mention that, in addition to affecting overall performance, internet addiction has significant effects on students’ academic performance.^7^, ^15^, ^16^, ^17^, ^18^, ^19^, ^20^, ^21^, ^22^

Several studies confirmed that students addicted to the Internet have significantly poor academic performance.^22^, ^23^, ^24^

Researchers have suggested in different studies that young people in different societies, especially university students, are at a higher risk for IA due to their social, environmental and educational conditions.^2^, ^24^ Several factors have been attributed to this vulnerability; first, students have more time to use the Internet than working people; second, students have the possibility of having unlimited access to Wi‐Fi related to the university systems, and of course, in many cases, students are encouraged and guided by their professors to use the Internet for research purposes.; third, being from the young generation, they tend to use the most up‐to‐date technologies. fourth, due to the circumstances and sometimes distance from the family, students are under less restrictions and censorship; fifth, being a student and, of course, being young requires diversity and one's own developmental interests.^25^

Until now, researchers have done much research to identify the underlying, igniting, and perpetuating factors of Internet addiction, and hypotheses have been proposed about individual, family, cultural, and social factors.^23^, ^24^, ^25^, ^26^, ^27^

Family performance is defined as a measure of the excellence of the physical and mental prosperity of all family members. This index includes five branches in a model called the Apgar family index: adaptation, participation, growth, affection, and determination.^27^, ^28^ The study on Chinese people reported that the degree of family functionality affects general adolescent behavior; it showed that their dependency on the Internet and drug use and their heavy and complicated alcohol consumption are related to their poor family functioning.^28^ Based on some other research, it seems that adolescents whose families have poor family functioning have inappropriate family relationships and less support and resources than their peers.^29^

An individual's perceived psychological or material resources in their immediate surroundings, such as family, friends, or other important individuals, are referred to as social support.^1^, ^23^ Research has shown that the level of perceived social support for individuals has positive effects on social relationships, emotional health, and well‐being. Those who did not have proper social support faced negative consequences in different aspects of their daily live.^24^ Students receiving support from parents and teachers showed increased positive academic and social performance. Furthermore, in the absence of social support from peers and family, teenagers struggled with academic success and communication. One may argue that teenagers’ perceptions of social support have a social and emotional role and that they also have an impact on lowering Internet addiction and other addictive behaviors.^1^, ^2^

Prior research has indicated a detrimental correlation between internet addiction and mental health in adolescents.^2^, ^21^ Overuse of the Internet causes mental health issues, psychological harm, and other health issues.^21^, ^22^, ^25^ Experts have recommended taking the required steps to stop internet addiction, figuring out how to lessen people's reliance on it, and, of course, promptly screening for the illness and its effects.^27^, ^28^, ^29^ On the other hand, one of the inevitable problems and changes during the spread of the Corona virus is maintaining distance in people's relationships. This distance in any type of interaction, including between colleagues in an office, students and teachers of schools, social interactions, and sometimes even in interaction between family members, is needed.^30^, ^31^

Adhering to adequate physical separation involves maintaining a distance of 6 feet from others during face‐to‐face interactions. On the other hand, social distancing entails remaining at home and limiting outside activities, which leads individuals to communicate virtually. The quarantine has several repercussions that require addressing individuals’ mental well‐being, such as the manifestation of dread and worry, melancholy and restlessness, sleep disruptions, anger, and even aggressiveness.^32^, ^33^, ^34^, ^35^, ^36^, ^37^, ^38^ Another significant consequence of quarantine is the reliance on the Internet for remote task management and obtaining information about the disease, including its prevalence, risks, and mortality. This increased exposure to information can potentially instill fear of the coronavirus. However, it is important to note that there is a risk of receiving inaccurate information from unreliable sources.^39^, ^40^

In addition to the global concern surrounding the COVID‐19 pandemic, the Internet and virtual space have emerged as a significant and integral part of people's lives, potentially leading to the global spread of Internet addiction.^41^

### 1.1 Knowledge gap and aims of the study

Therefore, due to the global challenge of internet addiction and its consequences, there has been limited research on the subject.^42^, ^43^, ^44^, ^45^, ^46^ Specifically, no previous study has comprehensively examined the relationship between probable risk factors in university students, one of the most vulnerable groups, particularly in the context of the COVID‐19 pandemic, which has the potential to fundamentally alter people's lifestyles. Our goal was to investigate internet addiction and the associated individual and societal risk factors among university students. Unlike previous studies, our study is novel and prioritized, offering a comprehensive approach to internet addiction.

## 2 METHODS

### 2.1 Study design

At the University of Medical Sciences in North Iran, a cross‐sectional analytical investigation was carried out. First, all 4110 students of the University of Medical Sciences were divided into groups (Medicinal, Midwife & Nurse, Dentistry, Rehabilitation Medicine), and then random sampling was done from each of these groups, and 318 students were selected. Examined and approved by the institutional ethics committees were the informed consent forms, patient recruiting materials, and study protocol. Under the guidelines of the code of ethics (IR. MUBABOL. REC.1400.019), the Ethics Committee of Babol University of Medical Sciences, Babol, Iran, authorized the study's first proposal. Data collection was done during August and September 2021. Written informed consent was given by the executors of the study to all participants. The forms followed the ethical guidelines outlined in the Declaration of Helsinki and were based on local regulatory needs. All patient demographic data was provided to the executors while maintaining confidentiality.

### 2.2 Study population and sampling

The sample consisted of 318 university students selected from among 4110 students in different fields. The statistical specialist's research reached the sample size using the formula with α = 0.05 and a0.31 estimation ratio, and an error rate of 5% through the random cluster sampling method. First, all 4110 students of the University of Medical Sciences were divided into groups (Medicinal, Midwife & Nurse, Dentistry, Rehabilitation Medicine), and then random sampling was done from each of these groups, and 318 students were selected. The objectives of the study were explained to the students, and they answered the prepared questionnaire as a one‐time link. In this research (Age/Gender/Marital status/field of study/place of living/smoke and alcohol use/fear of COVID‐19/Family APGAR/Family mental health) are independent variables, and Internet addiction is a dependent variable. Psychological distress and social support are composite variables.

### 2.3 Eligibility criteria

Inclusion criteria included spending at least one semester at the university and enrollment satisfaction. Questionnaires that were vaguely filled out and distorted were excluded from the study.

### 2.4 Study tools

The questionnaires included demographic information about participants, including their age, gender, marital status, education, residence, etc., as well as the tools, which we have described below.

- **Internet addiction test (IAT)**
  This questionnaire, originally devised by Dr. Kimberly Young, is one of the most authoritative in the area of Internet addiction. It consists of 20 items scored on a 5‐point Likert scale (1 = seldom, 2 = sometimes, 3 = often, 4 = often, and 5 = always). Each person's scores were categorized into three groups: 1. The average Internet user (scores 20–49); 2. The user who is experiencing issues due to excessive usage (scores 50–79); 3. The addicted user who is hooked on overuse and requires treatment (scores 80–100). The reliability and validity of the Persian translation were found to be appropriate.^47^
- **Fear of Coronavirus‐19 Scale (FCV‐19s)**
  Daniel Kwasi Ahorsu created a seven‐item measure called the Fear of Coronavirus‐19 Scale (FCV‐19S). A five‐point Likert scale is part of the survey. There is a minimum of 1 and a maximum of 5 for every question. Summarizing all of the scores (ranging from 7 to 35) yields the final score. The reliability and validity of the questionnaire in Iran have also been examined, and it has been reported that FCV‐19S is appropriate for assessing the psychological issues caused by COVID‐19.^48^, ^49^, ^50^, ^51^, ^52^
- **Family Apgar Scale**
  The Family Apgar Scale, first developed by Gabriel Smilkstein in 1987, is a tool to assess family status by healthcare workers. This questionnaire consists of 5 questions (adaptation‐participation‐emotional state development–problem solving, and decision making). A score of 7–10 indicates excellent family performance; a score of 4–6 indicates average family performance; and a score of 0–3 indicates poor family performance. The reliability and validity of the Persian version were reported to be appropriate.^53^
- **Fleming Social Support**
  The Fleming Social Support Scale has 25 questions and 5 sub‐scales of support from family, relatives, peers, general support, and beliefs about support. In the final form of this scale, the sub‐scales related to the support of friends and peers have been combined into a single subscale. The score range is 0–25, and a higher score indicates more support. It has acceptable validity and reliability in the Iranian population.^54^
- **Brief Symptom Inventory‐18 (BSI‐18)**
  This self‐assessment is a shortened form of BSI consisting of 53 sections, classified on nine scales. This questionnaire is the latest version of the tools designed by Derogatis and includes a description of 18 physical and emotional complaints. Participants rate their complaints on a scale of 0 (not at all) to 4 (very high). To date, methods grounded on factor analysis and classical test theory (CTT) have been used to study the dimensions of BSI‐18. Its Persian translation was judged to have acceptable validity and trustworthiness.^55^

### 2.5 Statistical analysis

The data were analyzed using SPSS software version 22. We used the standard deviation for quantitative and frequency data and the ratio for qualitative data. We also employed correlation analysis to investigate the relationship between Internet addiction and other factors. Multivariate linear regression was used to investigate the factors affecting Internet addiction. The level of significance was at *p* < 0.05.

## 3 RESULTS

The present study aimed to assess the relationship between internet addiction and emotional symptoms, fear of coronavirus, family performance, and social support during the COVID‐19 pandemic. The sample consisted of 318 university students selected from among 4110 students in different fields through randomized cluster sampling. All of them fulfilled the demographic and all other questionnaires clearly and concisely.

Based on our research, most of the participants (191/318, 60.1%) were female and 57.23% of them had doctorate level. The prevalence of Internet addiction based on the Internet Addiction Test (IAT ≥ 80) was 28.9% (92/318).

As shown in Table 1, the prevalence of internet addiction is significantly higher in women (64/191, 33.5%) compared with men (28/127, 22%). Also, the prevalence of internet addiction is significantly higher in students with a doctoral level of education (60/182, 32.9%) compared with a bachelor of sciences degree (32/136, 23.5%). However, there was no significant difference between the prevalence of internet addiction and the place of residence (dormitory vs. private house), age of students ( ≥ 21 vs. 22–23 years), and use of alcohol/cigarettes.

**Table 1:** Characteristics of the participants and Prevalence of internet addiction based on the demographic characteristic.

Variables		Internet addiction^a^		*p*‐value
	Number (%)	Yes	No	
		Number (%)	Number (%)	
Age	**(mean ± SD)**			0.341
≥21	21.6 ± 1.9	45 (48.9)	118 (52.2)	
22–31		47 (51.1)	108 (47.8)	
Place of living				0.258
Dormitory	68 (21.4)	17 (18.5)	51 (22.6)	
Private house	250 (78.6)	75 (81.5)	175 (77.4)	
Gender				0.018
Male	127 (39.9)	28 (30.4)	99 (43.8)	
Female	191 (60.1)	64 (69.6)	127 (56.2)	
Degree of education				0.043
Doctor	182 (57.23)	60 (65.2)	122 (54.0)	
Bachelor science	136 (42.76)	32 (34.8)	104 (46.0)	
Marital status				0.151
Single	295 (92.8)	88 (95.7)	207 (91.6)	
Married	23 (7.2)	4 (4.3)	19 (8.4)	
Use of alcohol **& cigarette**				0.266
No	290 (94.15)	82 (89.1)	208 (92)	
Yes	28 (5.85)	10 (10.9)	18 (8.0)	

a Internet addiction test (yes = IAT ≥ 80, no = IAT < 80).

Table 2 shows the mean scores of mental symptoms and their associated factors in students with internet addiction. Students addicted to the Internet had significantly higher mean scores of depression, anxiety, somatization (*p*‐value <0.001, <0.001, 0.004), and Global Severity Symptom Index (GSI) (*p*‐value <0.001). Additionally, students with IA had a higher mean score of fear of COVID‐19 (*p* = 0.003). However, there was no difference between students with and without IA in terms of the mean score of overall social support and its sub‐scales except for opinion to support, which had substantially lower ratings among students addicted to the Internet (*p* = 0.009).

**Table 2:** Profile of internet addiction of students based on psychological variables.

	Internet addiction			Total population
	No	Yes	*p*‐value	Mean ± SD
Variables	Mean ± SD	Mean ± SD		
Brief Symptom Inventory‐18				
Depression	4.7 ± 4.3	8.3 ± 5.7	˂0.001	5.7 ± 5.0
Anxiety	3.4 ± 4.1	5.7 ± 5.4	˂0.001	4.1 ± 4.6
Somatization	2.5 ± 2.9	3.9 ± 4.1	0.004	2.9 ± 3.3
GSI (Overall Psychological Distress)	0.6 ± 0.5	1.0 ± 0.7	˂0.001	0.7 ± 0.6
APGAR family support	7.0 ± 2.8	5.6 ± 2.8	˂0.001	6.6 ± 2.9
Social support				
Family relations	4.1 ± 2.0	3.9 ± 2.1	0.414	4.1 ± 2.0
Friends relations	5.5 ± 1.8	5.2 ± 1.7	0.175	5.4 ± 1.8
Opinion relations	3.9 ± 1.2	3.5 ± 1.3	0.009	3.8 ± 1.3
General relations	3.7 ± 1.0	3.9 ± 0.9	0.373	3.8 ± 1.0
Total score	17.4 ± 4.1	16.6 ± 4.3	0.111	17.1 ± 4.2
Fear of covid‐19	14.8 ± 5.3	17.1 ± 6.3	0.003	15.5 ± 5.7

Table 3 shows the risk factors of internet addiction in Medical Sciences students based on a multiple linear regression model. The results revealed that women are at a significant risk of internet addiction twice men (*p* = 0.028). Also, students who were in the medicinal field *p* = 0.043(, student with positive history of mental illness in family) *p* = 0.001 (and students with fear of COVID‐19 (*p* = 0.002) are at a significant risk for Internet addiction. However, marital status, place of living, total social support are not risk factors for internet addiction. The results confirmed that depression)*p* < 0.001(, anxiety) *p* < 0.001(, somatization) *p* = 0.001 and psychological distress)*p* < 0.001 are risk factors of internet addiction in the students. However, the total score of family APGAR is a protective factor (*p* < 0.001) against internet addiction.

**Table 3:** Risk factors of internet addiction in Iranian Medical Sciences students.

Variables			Crude OR	95% confidence interval	*p*‐value	Adjusted OR	95% confidence interval	*p*‐value
Age			0.681	0.858–1.105	0.681			
Gender (male)			0.561	0.335–0.940	**0.028**			
Marital status (single)			2.019	0.668–6.107	0.213			
Field of study		Medicine	2.026	1.023–4.016	**0.043**			
		Midwifery/Nurse	1.584	0.667–3.764	0.298			
		Dentistry	2.071	0.884–4.853	0.094			
		Rehabilitation	1.726	0.522–5.705	0.371			
place of living (home)			1.286	0.697–2.371	0.421			
Smoke & alcohol			0.710	0.314–1.602	0.409			
History of mental health in family			0.310	0.158–0.610	**0.001**			
Fear of covid‐19			1.070	1.026–1.116	**0.002**	1.055	1.007–1.106	**0.024**
Social support	Family		0.952	0.846–1.071	0.413			
	Friends		0.915	0.805–1.040	0.176			
	Opinion		0.791	0.662–0.945	**0.010**			
	General		1.108	0.875–1.404	0.394			
	Total		0.955	0.902–1.011	0.111			
Total score of family APGAR			0.852	0.784–0.926	**˂0.001**	0.900	0.817–0.992	**0.033**
BSI‐18 Brief Symptom Inventory‐18	Somatization		1.122	1.046–1.203	**0.001**			
	Depression		1.145	1.089–1.204	**˂0.001**	1.104	1.043–1.167	**0.001**
	Anxiety		1.106	1.050–1.164	**˂0.001**			
	Total score		1.053	1.030–1.076	**˂0.001**			
Overall Psychological distress (GSI)			2.517	1.708–3.707	**˂0.001**			

*Note*: Bold values are statistically significant.

## 4 DISCUSSION

This cross‐sectional analytical investigation was undertaken in northern Iran. The sample consisted of 318 university students chosen at random using the cluster sampling approach. The purpose of this study was to look at the key risk variables for Internet addiction among university students during the COVID‐19 epidemic. The significance of Internet addiction and reliance, as well as its prevalence in many communities, is not lost on anybody today, particularly among students, who are considered a vulnerable demographic group. In this research, we took a thorough approach to the topic. According to our research, the prevalence of Internet addiction is 28.9%. According to our findings, women are twice as likely as men to develop Internet addiction. Furthermore, students in the medical sector, students with a positive family history of mental illness, and students who are afraid of COVID‐19 are identified as having a high risk of Internet addiction. Thus, sadness, somatization, and psychological discomfort are risk factors for Internet addiction among students. However, age, marital status, location of residence, complete social support, and usage of alcohol or cigarettes are not risk factors for Internet addiction. Our result is congruent with the research findings of Schafer JO et al.^43^ who indicated that depression, anxiety, and interpersonal sensitivity were connected with Internet addiction, and poor self‐esteem was associated with potential Internet users. However, they didn't assess the role of fear of COVID‐19 and family roles, which is inconsistent with our study. This point is worth noting. Considering the epidemic of COVID‐19 and its impact on different areas of people's lives, it is important to be specially considered and evaluated in studies, and this is one of the important superiorities of our study. In relation to considering the role of the family, of course, considering the cultural differences in different societies, it is still very important to be discussed in different studies. Cynthia Sau Ting Wu et al. investigated parenting approaches, family functionality, and internet addiction among adolescents.^28^ L, they stated that 25.3% of the adolescent respondents exhibited IA; it was 28.9% of university students in our study. Additionally, they noted that some families—such as divorced or low‐income families, families with family conflict, and very dysfunctional families—were positively predictive of IA in teenagers. Contrary to our study, they didn't assess the family history of psychiatric disorders and their impact on IA. We found that a positive familial history of mental disorders has a significant role in the IA of university students. One of the issues that is always important in psychiatry is the role of genetics and inheritance in psychiatric disorders. This is a strong and important superiority of our study compared to their research, because the family history of psychiatric problems needs special attention. In line with our findings on the influence of COVID‐19 on internet addiction in students, Reihaneh Moniri et al.^30^ revealed that anxiety and dread of COVID‐19 had a positive and significant link with both Internet addiction and maladaptive cognitive emotion regulation techniques. However, they didn't assess other risk factors, such as a comprehensive approach, which was inconsistent with our study. The finding of Zenebe et al.^23^ regarding social support and internet addiction showed the significant negative effects of social support on Internet addiction, which was in contrast with our study result. It can be due to the discrepancy in the questionnaires and Chinese culture versus Iranian people. Because the way of providing social support in different societies is related to many factors and based on social, economic, cultural, political, and insurance differences, the amount and way of providing it are different, and as mentioned, the difference between the results of our study and their research can be related to the mentioned factors.

In this research, we attempted to have a holistic approach to the topic of Internet addiction and its associated aspects in the university student population, which was the superiority and uniqueness of our work. However, due to the impossibility of clinical psychiatry interviews based on DSM‐5 for assessing emotional symptomatic comorbidity disorders, we used questionnaires because of the COVID‐19 pandemic disease and its curfew, and in fact, not using a clinical interview based on DSM‐5 has been a limitation of our study. As a result, we suggested performing comparable studies assessing psychiatric comorbidities based on DSM‐5 clinical interviews in the future to support the validity of the data report.

## 5 CONCLUSION

The findings of our study showed a high and noteworthy prevalence of Internet addiction in students, especially in women and in people with doctoral education. Our results also showed a significant role of depression, anxiety, somatization, psychological distress, fear of COVID‐19, and a positive history of mental illness in family members in Internet addiction in university students, particularly in the medicinal field. However, age, marital status, place of living, and total social support are not risk factors for internet addiction. Therefore, it is necessary to pay special attention to students with these risk factors to reduce Internet addiction and its consequences in them, and our findings show that, considering the high prevalence of Internet addiction disorder in students, planning for timely screening and diagnosis and then appropriate referral to psychiatric services is required to receive the necessary treatments, including psychotherapy and drug treatment.

## AUTHOR CONTRIBUTIONS

**Mahbobeh Faramarzi:** Conceptualization; Investigation; Supervision; Visualization. **Bardia Karim:** Data curation. **Zeinab Gholami:** Data curation. **Fatemah Khoshkhou:** Data curation. **Fatemeh Shirazi:** Data curation. **Negar Soghli:** Data curation. **Munire Parvaneh:** Data curation; Conceptualization; Project administration. **Hoda Shirafkan:** Methodology; Software; Formal analysis. **Faezeh Khorshidian:** Conceptualization; Writing—original draft; Writing—review and editing; Visualization.

## CONFLICT OF INTERESTS STATEMENT

The authors declare no conflictS of interest.

## ETHICAL STATEMENT

The initial proposal of this study was approved by the Ethics Committee of Babol University of Medical Sciences, Babol, Iran, by the code of ethics: (IR. MUBABOL. REC.1400.019).

## INFORMED CONSENT

before the study, the objectives of the study were fully explained to the volunteers, and informed consent was obtained from all participants of the study.

## TRANSPARENCY STATEMENT

The lead author Faezeh Khorshidian affirms that this manuscript is an honest, accurate, and transparent account of the study being reported; that no important aspects of the study have been omitted; and that any discrepancies from the study as planned (and, if relevant, registered) have been explained.

## Data Availability

The data that support the findings of this study are openly available in Faezeh khorshidian at Endnote, reference number 30. All the data and materials of this study are available. To access the data, the corresponding author(F. KHO)of this article should be contacted.
